# Anion inhibition studies of the Zn(II)-bound ι-carbonic anhydrase from the Gram-negative bacterium *Burkholderia territorii*

**DOI:** 10.1080/14756366.2020.1867122

**Published:** 2021-01-04

**Authors:** Andrea Petreni, Viviana De Luca, Andrea Scaloni, Alessio Nocentini, Clemente Capasso, Claudiu T. Supuran

**Affiliations:** a Department of NEUROFARBA, Section of Pharmaceutical and Nutraceutical Sciences, University of Florence, Polo Scientifico, Firenze, Italy; b Department of Biology, Agriculture and Food Sciences, CNR, Institute of Biosciences and Bioresources, Napoli, Italy; c Proteomics and Mass Spectrometry Laboratory, ISPAAM, CNR, Naples, Italy

**Keywords:** Carbonic anhydrase, ι-class, anion, inhibitor, *Burkholderia territorii*

## Abstract

*Burkholderia territorii*, a Gram-negative bacterium, encodes for the ι-class carbonic anhydrase (CA, EC 4.2.1.1) BteCAι, which was recently characterised. It acts as a good catalyst for the hydration of CO_2_ to bicarbonate and protons, with a k_cat_ value of 3.0 × 10^5^ s^−1^ and k_cat_/K_M_ value of 3.9 × 10^7^ M^−1^ s^−1^. No inhibition data on this new class of enzymes are available to date. We report here an anion and small molecules inhibition study of BteCAι, which we prove to be a zinc(II)- and not manganese(II)-containing enzyme, as reported for diatom ι-CAs. The best inhibitors were sulphamic acid, stannate, phenylarsonic acid, phenylboronic acid and sulfamide (K_I_ values of 6.2–94 µM), whereas diethyldithiocarbamate, tellurate, selenate, bicarbonate and cyanate were submillimolar inhibitors (K_I_ values of 0.71–0.94 mM). The halides (except iodide), thiocyanate, nitrite, nitrate, carbonate, bisulphite, sulphate, hydrogensulfide, peroxydisulfate, selenocyanate, fluorosulfonate and trithiocarbonate showed K_I_ values in the range of 3.1–9.3 mM.

## 1. Introduction

The carbonic anhydrase (CA, EC 4.2.1.1) superfamily is composed nowadays of eight genetically distinct classes, the α-, β-, γ-, δ-, ζ-, η-, θ-, and ι-CAs, which probably may soon increase when additional families will be reported in other organisms. All of them catalyse a simple but physiologically crucial reaction, the carbon dioxide reversible hydration to bicarbonate and protons CO_2_ + H_2_O ⇄ HCO_3_^−^ + H^+.^^1–6^. CO_2_ hydration/bicarbonate dehydration are relevant in organisms all over the phylogenetic tree, from simple to complex ones. In most cells, tissues and organs, these enzymes participate in crucial physiologic processes connected to pH regulation, metabolism, secretion of electrolytes, transport of gases and anions, and others, which are also therapeutically relevant (at least for mammalians)^5^^,^^7^^,^^8^. In fact, both CA inhibitors (CAIs)^5–8^, and CA activators (CAAs)^9^^,^^10^ have many therapeutic applications in a variety of fields, starting with diuretics and antiglaucoma agents and ending with anticancer/antimetastatic drugs (for the inhibitors)^5–8^, but also including memory therapy, modulation of emotional memory and fear extinction memory activator agents^9^^,^^10^. Recently, inhibition of CAs from pathogenic organisms has also been proposed as an innovative approach to develop anti-infectives, which may target bacterial infections resistant to clinically used antibiotics^4^^,^^11–13^, but also to treat protozoan-provoked^14^^,^^15^ as well as fungal infections^16^^,^^17^. Indeed, various classes of inhibitors were shown to be effective in a variety of models^4^^,^^11–17^, which inspired researchers to find novel chemotypes acting as modulators of activity as well as novel potential drug targets^4^^,^^11–17^.

Very recently, a gene coding for a member of the ι-CA family has been originally described to occur in the genome of the marine diatom *Thalassiosira pseudonana*^2^^a^; the corresponding enzyme has been isolated and reported preferring Mn(II) as a metal cofactor in its active site, and not Zn(II) frequently found therein in other organisms. In the same paper, it has been shown that members of the ι-CA family should be present also in bacteria, as deduced by genome analysis, although such enzymes were not characterised at that moment in such organisms. Recently, we confirmed the finding of Gontero’s group^2^^a^, and reported the cloning and biochemical characterisation of the first example of a bacterial ι-CA, which was observed in the Gram-negative bacterium *Burkholderia territorii* and denominated BteCAι^2^^b^. The enzyme showed a significant CO_2_ hydrase activity, with kinetic parameters (k_cat_ of 3.0 × 10^5^ s^−1^ and k_cat_/K_M_ of 3.9 × 10^7^ M^−1^ s^−1^) comparable to those of highly efficient bacterial and even mammalian isoforms, such as human (h) CA I^2^^b^. However, no inhibition studies have been performed so far on this enzyme, which has been demonstrated to be dimeric (by using protonography)^18^ and also to be a zinc- and not manganese-dependent enzyme^2^^b^. Here, we prove that this is indeed the case by using atomic absorption spectroscopy, and also report the first inhibition study of the enzyme, with small molecules and anions, a well-known class of CAIs^19^.

## 2. Materials and methods

### 2.1. Chemistry

Anions and small molecules were commercially available reagents of the highest available purity from Sigma-Aldrich (Milan, Italy). Purity of tested compounds was higher than 99%.

### 2.2. Atomic absorption spectrometry

In various enzyme samples, the content of Zn(II) and Mn(II) was measured with a flame PinAAcle 500 Perkin Elmer instrument, located in the Interdepartmental Service Centre for Biotechnology of Agricultural, Chemical and Industrial Interest (CIBIACI), University of Florence.

### 2.3. Enzymology

BteCAι was a recombinant enzyme obtained in-house as described earlier^2^^b^.

### 2.4. Ca catalytic activity and inhibition assay

An Applied Photophysics stopped-flow instrument has been used for assaying the CA catalysed CO_2_ hydration activity^20^. Phenol red (at a concentration of 0.2 mM) has been used as an indicator, working at the absorbance maximum of 557 nm, with 10–20 mM HEPES (pH 7.5) as buffers, and 20 mM NaClO_4_ (for maintaining constant the ionic strength), following the initial rates of the CA-catalysed CO_2_ hydration reaction for a period of 10–100 s. The CO_2_ concentrations ranged from 1.7 to 17 mM for the determination of the kinetic parameters and inhibition constants. For each inhibitor, at least six traces of the initial 5–10% of the reaction were used to determine the initial velocity. The uncatalyzed rates were determined in the same manner and subtracted from the total observed rates. Stock solutions of inhibitors (10 mM) and dilutions up to 0.01 µM were prepared in distilled-deionised water. Inhibitor and enzyme solutions of concentrations ranging between 5 and 10 nM were preincubated together for 15 min, at room temperature, prior to assay, in order to allow for the E-I complex formation. The inhibition constants were obtained by non-linear least-squares methods using PRISM 3 and the Cheng-Prusoff equation, as reported earlier^21^, and represent the mean from at least three different determinations.

## 3. Results and discussion

Gontero’s group reported that the ι-CA isolated from the marine diatom *T. pseudonana*^2^^a^ is active with Mn(II) bound as a metal cofactor within its active site and not with Zn(II), such as most other CA isoforms known to date. However, it should be mentioned that γ-CAs are active with Fe(II)^22^ and ζ-CAs with Cd(II)^23^ present at their active sites, as well as with Zn(II), so that the use of alternative metal ions to zinc is not improbable. Thus, we prepared the recombinant BteCAι as described earlier^2^^b^, both in the presence of zinc as well as manganese salts in order to assay which of the two metal ions are incorporated into the holoenzyme. As seen from Table 1, significant amounts of Zn(II) were found in all protein samples investigated, with trace quantities of Mn(II). The amount of Mn(II) was the same in both enzyme samples, even those prepared in the presence of high concentrations of Mn(II) salts (possibly due to contaminants in the buffers/reagents used to prepare the enzyme). The content of zinc ion per polypeptide chain was determined as 1:1 (within experimental error limits). Hence, unlike the diatom enzyme^2^^a^, the bacterial ι-CA was proved to be a zinc-containing enzyme.

**Table 1.** Percentage of zinc and manganese in BteCAι as determined by atomic absorption spectroscopy.

Enzyme	Concentration (µM)	% Zn-BteCAι	% Mn-BteCAι
Zn-BteCAι – sample 1	33.5	98.30	1.70
Zn-BteCAι – sample 2	46.6	99.93	0.07
Mn-BteCAι – sample 1	20.0	97.85	2.15
Mn-BteCAι – sample 2	21.0	99.74	0.26

The buffer used for sample preparation reported 7.0 × 10^−3 ^ppm of Zn and 1.1 × 10^−2 ^ppm of Mn.

We also investigated the inhibition of the bacterial enzyme BteCAι with a wide range of inorganic anions and small molecule compounds known to interact with the CA family of proteins (Table 2)^19^. Although anion inhibitors are usually not highly effective, they are relevant both for understanding in detail the inhibition mechanisms of metalloenzymes and for drug design purposes; this was the reason why many CAs belonging to various families were profiled for their inhibition with anions^19^.

**Table 2.** Anion inhibition data of BteCAι as determined by a stopped-flow CO_2_ hydrase assay.^20^

Anion	K_I_ (mM)^a^			
	hCA I^b^	hCA II^b^	*E. coli* β-CA^c^	BteCAι^d^
F^−^	>300	>300	9.4	4.6
Cl^−^	6	200	6.7	3.1
Br^−^	4	63	3.8	4.8
I^−^	0.3	26	>50	>50
CNO^−^	0.0007	0.03	0.58	0.79
SCN^−^	0.2	1.6	5.7	6.1
CN^−^	0.0005	0.02	>50	>50
N_3_^−^	0.0012	1.51	>50	>50
NO_2_^−^	8.4	63	4.9	8.4
NO_3_^−^	7	35	2.4	6.2
HCO_3_^−^	12	85	0.81	0.94
CO_3_^2−^	15	73	0.89	4.4
HSO_3_^−^	18	89	3.7	8.4
SO_4_^2−^	63	>200	1.7	5.8
HS^−^	0.0006	0.04	2.7	6.2
NH_2_SO_2_NH_2_	0.31	1.13	0.011	0.086
NH_2_SO_3_H	0.021	0.39	0.0025	0.0062
PhAsO_3_H_2_	31.7	49	0.0061	0.008
PhB(OH)_2_	58.6	23	0.0028	0.009
ClO_4_^−^	>200	>200	>50	>50
SnO_3_^2−^	0.57	0.83	0.52	0.094
SeO_4_^2−^	118	112	3.1	0.73
TeO_4_^2−^	0.66	0.92	0.51	0,71
OsO_5_^2−^	0.92	0.95	>50	>50
P_2_O_7_^2−^	25.8	48	>50	>50
V_2_O_7_^2−^	0.54	0.57	>50	>50
B_4_O_7_^2−^	0.64	0.95	0.25	>50
ReO_4_^−^	0.11	0.75	>50	>50
RuO_4_^−^	0.101	0.69	9.5	>50
S_2_O_8_^2−^	0.107	0.084	6.4	7.4
SeCN^−^	0.085	0.086	3.1	6.6
NH(SO_3_)_2_^2−^	0.31	0.76	1.5	>50
FSO_3_^−^	0.79	0.46	0.83	9.3
CS_3_^2−^	0.0087	0.0088	3.1	8.6
EtNCS_2_^−^	0.00079	0.0031	0.084	0.81
PF_6_^−^	>50	>50	>50	>50
CF_3_SO_3_^−^	>50	>50	>50	>50

Inhibition of the human isoforms hCA I and II, and the bacterial β-CA from *Escherichia coli* are also shown for comparison.

^a^Mean from 3 different assays, by a stopped flow technique (errors were in the range of ± 5–10% of the reported values).

^b^From Ref. ^19^.

^c^From Ref. ^24^.

^d^This work.

The data of Table 2 show the following interesting aspects for the inhibition of this poorly investigated CA class:

- some anions, among which iodide, cyanide, azide, perchlorate, perosmate, diphosphate, divanadate, tetraborate, perrhenate, perruthenate, iminodisulfonate, hexafluorophosphate and trifluoromethanesulfonate, did not inhibit BteCAι significantly up to 50 mM concentration of inhibitor in the assay system. This is not unexpected for anions with low affinity for complexing metal ions, such as perchlorate, hexafluorophosphate and trifluoromethanesulfonate^19^, but it is rather surprising for iodide, cyanide, and azide, which have quite a high affinity for metal ions in solution and in the active site of many metalloenzymes^19^. Indeed, some of these anions show a potent inhibitory action for other CAs, such as the isoform hCA I (Table 1 and Ref. ^19^). As no X-ray crystallographic data are available so far for ι-CAs, it is impossible to rationalise these interesting and surprising data.
- The following inhibitors showed inhibitory action against BteCAι in the millimolar range: fluoride, chloride, bromide, thiocyanate, nitrite, nitrate, carbonate, bisulphite, sulphate, hydrogensulfide, peroxydisulfate, selenocyanate, fluorosulfonate and trithiocarbonate (KI values in the range of 3.1–9.3 mM). As above, some of these data stupefied us: sulphate, for example, is a highly inefficient inhibitor of many α-CAs (e.g. hCA I and II), but it inhibits efficiently the bacterial β- and ι-CAs shown in Table 1.
- Even more efficient inhibitory action against BteCAι was registered for the following anions: diethyldithiocarbamate, tellurate, selenate, bicarbonate and cyanate, which were submillimolar inhibitors with KI values ranging between 0.71 and 0.94 mM (Table 1). The bicarbonate high affinity is of interest, since this anion is also a substrate/reaction product of the CA – catalysed reactions.
- The most efficient BteCAι inhibitors detected so far were stannate, sulphamic acid, phenylarsonic acid, phenylboronic acid and sulfamide, with KI values of 6.2–94 µM (Table 1). Some of these compounds, such as sulfamide and sulphamic acid, act as effective inhibitors of many other CAs (for example, see the E. coli β-CA inhibition data shown in Table 1). They also inhibit the human isoforms hCA I and II (although to lower levels compared to the bacterial enzymes). The stannate data is also quite interesting. This anion is an order of magnitude better as a BteCAι inhibitor compared to its inhibition level of other CAs investigated so far.

## 4. Conclusions

We investigated the nature of the metal ion within the active site of the first bacterial ι-CA described so far, namely BteCAι, whose corresponding gene was found in the genome of the Gram-negative bacterium *B. territorii*. Unlike the diatom enzyme cloned from *T. pseudonana*, the bacterial ι-CA has Zn(II) ions at its active site and not Mn(II) counterparts. We also report here the first inhibition study of BteCAι with a range of inorganic anions and small molecules known to act as CA inhibitors. The most efficient BteCAι inhibitors were stannate, sulphamic acid, phenylarsonic acid, phenylboronic acid and sulfamide, with K_I_ values of 6.2–94 µM. Diethyldithiocarbamate, tellurate, selenate, bicarbonate and cyanate were submillimolar inhibitors, with K_I_s ranging between 0.71 and 0.94 mM. Fluoride, chloride, bromide, thiocyanate, nitrite, nitrate, carbonate, bisulphite, sulphate, hydrogensulfide, peroxydisulfate, selenocyanate, fluorosulfonate and trithiocarbonate showed K_I_ values in the range of 3.1–9.3 mM, whereas no inhibition was registered for iodide, cyanide, azide, perchlorate, perosmate, diphosphate, divanadate, tetraborate, perrhenate, perruthenate, iminodisulfonate, hexafluorophosphate and trifluoromethanesulfonate. These data may be useful for designing more efficient ι-CA inhibitors.
